# Midwives’ Experiences with and Perspectives on Online (Nutritional) Counselling and mHealth Applications for Pregnant Women; an Explorative Qualitative Study

**DOI:** 10.3390/ijerph18136733

**Published:** 2021-06-23

**Authors:** Renate F. Wit, Desiree A. Lucassen, Yvette H. Beulen, Janine P. M. Faessen, Marina Bos-de Vos, Johanna M. van Dongen, Edith J. M. Feskens, Annemarie Wagemakers, Elske M. Brouwer-Brolsma

**Affiliations:** 1 Division of Human Nutrition and Health, Department Agrotechnology and Food Sciences, Wageningen University and Research, P.O. Box 17, 6700 AA Wageningen, The Netherlands; renate.wit@wur.nl (R.F.W.); desiree.lucassen@wur.nl (D.A.L.); yvette.beulen@wur.nl (Y.H.B.); janine.faessen@wur.nl (J.P.M.F.); edith.feskens@wur.nl (E.J.M.F.); 2 Health and Society, Department of Social Sciences, Wageningen University and Research, P.O. Box 8130, 6700 EW Wageningen, The Netherlands; annemarie.wagemakers@wur.nl; 3 Faculty of Industrial Design Engineering, Delft University of Technology, Landbergstraat 15, 2628 CE Delft, The Netherlands; m.bos-devos@tudelft.nl; 4 Department of Health Sciences, Faculty of Science, Vrije Universiteit Amsterdam, Amsterdam Movement Sciences, De Boelelaan 1085, 1081 HV Amsterdam, The Netherlands; j.m.van.dongen@vu.nl

**Keywords:** healthcare professionals, mHealth gestation, maternal, health, nutrition, apps, midwives

## Abstract

Prenatal nutrition is a key predictor of early-life development. However, despite mass campaigns to stimulate healthy nutrition during pregnancy, the diet of Dutch pregnant women is often suboptimal. Innovative technologies offer an opportunity to develop tailored tools, which resulted in the release of various apps on healthy nutrition during pregnancy. As midwives act as primary contact for Dutch pregnant women, the goal was to explore the experiences and perspectives of midwives on (1) nutritional counselling during pregnancy, and (2) nutritional mHealth apps to support midwifery care. Analyses of eleven in-depth interviews indicated that nutritional counselling involved the referral to websites, a brochure, and an app developed by the Dutch Nutrition Centre. Midwives were aware of the existence of other nutritional mHealth apps but felt uncertain about their trustworthiness. Nevertheless, midwives were open towards the implementation of new tools providing that these are trustworthy, accessible, user-friendly, personalised, scientifically sound, and contain easy-digestible information. Midwives stressed the need for guidelines for professionals on the implementation of new tools. Involving midwives early-on in the development of future nutritional mHealth apps may facilitate better alignment with the needs and preferences of end-users and professionals, and thus increase the likelihood of successful implementation in midwifery practice.

## 1. Introduction

Prenatal (i.e., placental) and postnatal (i.e., breastmilk or infant formula) substrate supply, including macro and micronutrients, antibodies, and hormones [1,2,3], are key predictors of early-life development and growth [1,4]. However, despite public health campaigns to promote healthy nutrition during pregnancy, food choices of Dutch women of reproductive age are still often suboptimal. Data of a Dutch cohort study including women with a pregnancy wish showed, for instance, inadequate intakes of the fish fatty acids EPA (Eicosapentaenoic acid) and DHA (Docosahexaenoic acid), vitamin D, and folic acid [5,6], which have all been associated with early-life (brain) development, cognitive- and psychological functioning in children [7,8,9,10,11]. More generally, maternal diet during pregnancy has been associated with an infant’s risk of developing non-communicable diseases in later life [1]. Thus, more effective strategies to encourage healthy food choices during pregnancy are urgently needed.

More personalised strategies to initiate dietary behaviour changes may be more effective than public health campaigns, and rapid evolvement of technological tools and mobile internet accessibility now offers the opportunity to provide such personalised counselling through mobile applications (i.e., apps) [12,13,14,15,16]. Nowadays, women are shifting from traditional sources toward digital sources for pregnancy-related information and support. For example, an Australian study showed that over 75% of Australian women used at least one pregnancy app [17]. These women mentioned they found pregnancy apps a useful information source, used them for self-monitoring of bodily changes, but also tracked foetal development [17]. Accordingly, the popularity of apps—also attributable to the perceived advantage in terms of cost-effectiveness, time-effectiveness, and labour-intensiveness—among health professionals, is increasing rapidly [14]. In fact, for pregnancy-specific nutrition information, even more apps have been developed than for any other medical topic [14,18].

However, to date, evidence on the overall benefit of nutritional mHealth apps for pregnant women is limited. Brown et al. [14] reviewed the availability and quality of Australian apps on healthy nutrition during pregnancy and showed that these apps were limited by the inclusion of only a small number of behaviour change techniques and limited quality of the nutritional content. Moreover, the actual effectiveness of mHealth apps (i.e., mobile healthcare apps, referring to phone, tablets, and wearables) on dietary behaviour change in pregnant women has only been investigated in a limited number of studies [19,20,21]. More specifically, six months of participation in an online personalised coaching platform—without professional guidance—effectively increased the intake of fruit and vegetable and decreased alcohol intake in pregnant couples or couples with a pregnancy wish [19]. Moreover, a randomised controlled trial among 565 overweight and obese pregnant women showed that receiving nutritional advice by means of an mHealth app and nutritionist/dietician resulted in a lower daily intake of energy, carbohydrates, sugars, fat, saturated fat and higher intake of protein compared to usual care [20]. In contrast, Dodd et al. [22] did not observe an additional benefit of a smartphone app as part of an extensive dietary, physical activity, and behavioural intervention comprising of face-to-face and telephone-based counselling by a dietician. Although all pregnant women showed an improved diet quality, only a limited number of women adopted use of the app. Despite these mixed findings with respect to dietary behaviour change among pregnant women, evidence on the potential benefit of mHealth apps as part of standard care is accumulating [21,23].

Until now, there is limited knowledge on the experiences and perspectives of midwives with respect to the implementation of mHealth apps in midwifery practice. Insights into this topic are important as midwives are the primary contact for pregnant women in the Netherlands. Hence, for a successful implementation of a nutritional mHealth app among pregnant women, the trust and support of midwives is of utmost importance. Therefore, we explored the experiences and perspectives of midwives on (1) nutritional counselling during pregnancy, and (2) strengths and limitations of nutritional mHealth apps to support midwifery care, using a theoretical framework approach.

## 2. Materials and Methods

### 2.1. Study Design

This qualitative study is conducted as part of the Regiodeal Foodvalley (incl. Wageningen) and its “Prille start” (Early-life) project, which aims to develop dietary coaching tools for pregnant and lactating women and their (unborn) children. To gain insights into the experiences and perspectives of midwives regarding nutritional mHealth apps for pregnant women, 11 in-depth interviews (±30 min) were conducted. Prior to the interviews, the topic list of the interview guide was compiled, which was based on the Theoretical Domains Framework (TDF) and COM-B model. The validated TDF, consisting of 14 domains, was designed to assist healthcare professionals (hereinafter referred to as professionals) to identify their behaviour on the implementation of evidence-based research in daily practices [24] and has been used as such in previous studies [25,26,27]. The COM-B model was designed to identify what needs to change for a behaviour change intervention to be effective and focuses on three components (i.e., ‘capability’, ‘opportunity’, and ‘motivation’).[28,29] Atkins et al. [28] integrated the relevant domains of the TDF in the COM-B model, which resulted in a combined framework that was used in this study.

### 2.2. Recruitment and Data Collection

All midwives employed in the Netherlands were eligible to participate in the study. A purposive sampling method was applied to recruit participants of various ages and working areas (urban, rural). Potential participants were approached either via a general email to a midwifery practice or via a personal email to a midwife within the network of the researchers. The recruitment and interviews took place between October 2020 and March 2021. Due to the Corona pandemic, interviews were conducted through online Zoom and MS Teams meetings while using audio and video. All participants were informed about the study purpose and provided written informed consent prior to participation. Data-saturation was assumed to be acquired within six to 12 interviews [30], which in this study appeared to be reached after eight interviews. To ensure data-saturation, three additional interviews were conducted as proposed by Francis et al. [31]. The interview guide (Supplement 1) was based on the integrated TDF-COM-B framework by Atkins et al. [28], and consisted of three parts. The first part was a factsheet to record all interview and participant details e.g., date, time, participant number, age, job, and working area. The second part included the interview questions. Questions were partly open-ended and aimed to explore midwives’ perspectives on and experiences with online (nutritional) counselling and the use of mHealth apps for pregnant women. Examples of questions were ‘*How do you feel about the flexibility of your own behaviour when it comes to improved technology in the form of apps for pregnant women?*’ and ‘*What features would you like to see in a future app?*’ It should be emphasised that we conducted in-depth interviews and that the interview guide served as guidance. More specifically, in certain cases, prepared questions were not applicable and as such not raised during the interview. An example of such a question is ‘*How do you feel about setting yourself a goal for your intentions to improve dietary intake in pregnant women or the use of an app to improve this?*’. The third part of the factsheet consisted of a post-interview comment sheet where the first author noted all emotions, questions, and other aspects that came up or were noticed during the interview. All interviews were performed, transcribed verbatim, and summarised by the primary researcher (R.F.W). Summaries were sent to the participants, as a member check, to assess whether the main message of their responses was interpreted correctly.

### 2.3. Analysis

To organise the data and identify patterns corresponding to the research question, a thematic content analysis was performed. Interviews were inductively coded by two researchers (R.F.W, J.P.M.F). The first two interviews were open coded by both researchers. Thereafter, codes were discussed until consensus was reached on the coding scheme. The remaining interviews were coded by both researchers using the coding scheme. To maintain focus on the research question, only relevant parts of the interview were coded. All codes were translated into English and discussed until consensus was reached (axial coding). If consensus was not reached, a senior researcher (AW) assisted in decision making and supervised the coding process. Subsequently, links were searched between the codes and corresponding codes were grouped into overarching themes and structured in a code tree (selective coding). The coding and analyses of the interviews were performed in MAXQDA 2020.

## 3. Results

A total of 11 midwives participated in the study (Table 1); all were women, and the mean age was 35 years (range 21–60 years). Midwives worked in primary care in solo or group practices (n = 8), as a clinical midwife in a hospital (n = 2) or both in primary care and in the hospital (n = 1). Two midwives worked part-time and pursued another profession. Interviews lasted on average 29 min and were conducted through Zoom (n = 10) and Microsoft Teams (n = 1).

**Figure A1:**
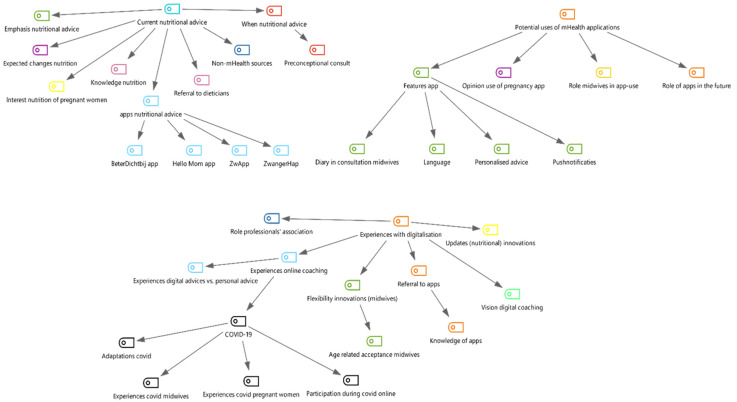
Code tree.

Three overarching themes were identified from the interviews: ‘Midwives and nutritional counselling,’ ‘Midwives’ experiences with mHealth’, and ‘Future of mHealth in midwifery practices’. The results are described according to these three themes. In the Appendix A, codes and sub-codes can be found in Table A1: Codes and sub-codes. Additionally, the code tree can be found in Figure A1: Code Tree.

### 3.1. Midwives and Nutritional Counselling

Nutritional counselling usually takes place during the pre-conceptional consult. This consult particularly focusses on a healthy lifestyle, and/or during the first consult around eight weeks of gestation (i.e., the intake). Most midwives discuss the relevance of a healthy diet during the intake by means of providing verbal nutritional advice, nutritional information through a brochure, and referral to the app/website of the Dutch Nutrition Centre. The brochure is called ‘Zwanger!’ and contains information about pregnancy and includes a small section about nutrition. This brochure was co-developed by several pregnancy and child health focussed institutes, including the professionals’ association of midwives in the Netherlands (KNOV). After the intake, nutrition is only discussed in case of medical necessity (e.g., because of extreme weight changes or disrupting lab results) or when women explicitly ask for more information. One midwife did not provide verbal nutritional information during the intake; she assumes a basic knowledge level and only provides nutritional advice when asked for. However, she does systematically refer all clients to the ZwangerHap app of the Dutch Nutrition Centre, which she considered trustworthy and always up to date. All midwives expressed their concerns about the focus of the nutritional advice for pregnant women in the Netherlands, i.e., being focussed on foods to be avoided rather than on a healthy dietary pattern. In their experience, the current recommendation discourages pregnant women to pursue a healthy diet, as the number of products advised against is rather high. Moreover, as the advice is quite strict and changes from time to time, some midwives feel unsure about the necessity of these recommendations. About half of the midwives explicitly said that it would be better to place more emphasis on the positive aspects of a health dietary pattern, rather than on all the discouraged foods.

Now the emphasis is on what you are not allowed to do and I find that annoying. ... with a few adjustments you can just do anything. I would like that, if it is approached in a much more positive way.

(P4)

Four midwives did not feel sufficiently knowledgeable to provide nutrition counselling, particularly in case of certain dietary needs. Two midwives also indicated lack of nutritional education, and six midwives did not feel capable to judge their clients’ diet quality from a nutritional diary. Another midwife identified the continuously changing nutritional perspectives as a bottleneck. Some midwives stated that they have not enough time to talk about nutrition; nutritional recommendations were considered too elaborate and proper nutritional counselling would require a separate consult. Finally, three midwives stated that clients should be referred to other healthcare professionals for dietary counselling, such as a nurse specialist or dietician. This is evidenced by the following quote:

Well, then you are actually taking over the work of a dietician, yes, and I think it would be better to leave that to the specialists.

(P9)

### 3.2. Midwives’ Experiences with mHealth

The current use of mHealth apps in midwifery practices is limited. All midwives refer to the ZwangerHap app of the Dutch Nutrition Centre as this is a very trustworthy source for nutritional advice in the Netherlands. Four midwives also knew about and sometimes refer to the ZwApp, which is a medical mHealth app developed in collaboration with midwives, gynaecologists, paediatricians, and nurses of a regional hospital. Two midwives mentioned the BeterDichtbij-app (BetterNearby-app), which is offered through hospitals and was developed by a collaboration of Dutch hospitals. Finally, one midwife mentioned the Hello Mom app, this app is not a nutritional app but used by midwives to hand over the digital echo pictures to their clients. The main reason for midwives to refer pregnant women to the ZwangerHap, and other future mHealth apps, would be to offer pregnant women the opportunity to independently access information beyond the consultations. However, when asked if they knew more about the ZwangerHap app, three midwives said that they had never opened the app themselves. During six interviews, there were speculations about the effectiveness of nutritional mHealth apps, i.e., whether nutritional knowledge (provided by an app) would really change the dietary behaviour of pregnant women. Even though all midwives were positive about the possibility of positive behaviour changes after gaining nutritional knowledge, only some of them said that a good app could play a role in increasing this knowledge. 

Two midwives had experiences with online coaching programmes for pregnant women, and both were positive about this. One midwife worked at a hospital where women can ask their questions via a chat system in the hospital’s app. The other midwife said that the quit smoking programmes for pregnant women, which involve online or telephonic coaching, worked well. However, not all midwives were positive about online nutritional information. Midwives felt that apps and websites do not always provide reliable information and the fact that their information is not always tailored to its users sometimes seemed to result in unnecessary stress among pregnant women. In addition, two midwives mentioned the importance of personal real-life contact, especially in case of vulnerable clients.

But in addition to this digitalisation, it is still very important that we continue to see the women and maintain physical contact. Especially the vulnerable group.

(P4)

About half of the midwives said there are too many digital innovations and new apps to keep up, and that they had not enough time to get familiar with these tools. Seven midwives pointed towards differences between younger and older professionals with regards to the use of mHealth. Whereas four midwives were not considering the use of mHealth apps, others did show their interest and thought that especially the younger generation of midwives would be open to using such tools. One midwife mentioned that there is always some resistance with the introduction of new strategies, guidelines, or tools. The midwives that worked in primary care indicated to receive professional news updates through newsletters of the KNOV. However, all but one did not recall having received (frequent) updates about recent digital innovations or new apps. As one of them noted:

I always read the newsletters from our professionals’ organisation, but I cannot recall how much has been said about new technology regarding nutrition in recent months.

(P5)

### 3.3. Future of mHealth in Midwifery Practices

Midwives were asked their opinion on the current use of mHealth apps. Several midwives mentioned that many pregnant women are interested in nutrition and appreciate the existence of such apps. However, for pregnant women needing more in-depth nutritional knowledge, the current apps were not considered suitable.

You just see that these educated women do things like this, and like and download apps like that, and of course that’s not the group where the greatest health gains can be made. And that is of course just really very difficult about these kinds of innovations and digital things. We just don’t get to the Syrian women in the disadvantaged neighbourhood, I really don’t see that happening in the next ten years and I find that poignant.

(P10)

The midwives believed that an app must be available in different languages and easy to understand language (n = 6), be interactive (n = 8), and there should be sufficient use of visuals, such as infographics (n = 3). Other features that were mentioned by the midwives included push notifications, automatic feedback on nutritional diaries, possibilities to personalise the app with e.g., medical information, cultural background, and budget. During the interviews, midwives were also asked for their opinion on the possibility of integrating a nutritional diary in the app. Most midwives were positive about this feature, but there was some resistance regarding the time it would cost pregnant women to complete the reporting and lack of time to discuss the diary during a consult. Most midwives believe mHealth apps are going to play a bigger role in daily life in the future and are willing to advise apps. All midwives, but one, were positive about the future role of online counselling in midwifery practice. For this midwife, she feared that it could lead to more loneliness in pregnant women when increasingly using digital tools in healthcare.

No, where my fear is, it is that for some people it can lead to more loneliness, indeed to a healthier diet, but to more loneliness.

(P11)

Overall, midwives are optimistic about this development and believe these mHealth apps can improve clients’ health as they are more involved with their own health and the health of their unborn child. Positive aspects that were mentioned about apps are the easy access provision to information, difficult topics that are explained in the app, and apps are fun to use in addition to regular care.

It’s just very easy to open the app instead of going all the way to Google.

(P5)

## 4. Discussion

In this study, we explored the experiences and perspectives of midwives on nutritional counselling and use of mHealth apps during gestation. Various barriers in the provision of nutritional counselling and promotion of mHealth apps were identified, including limited knowledge on healthy nutrition and limited capability to provide personalised nutritional counselling. Despite that, nearly all midwives discussed the general dietary guidelines during one or more individual counselling sessions. Other identified barriers included doubts on the trustworthiness of the apps and struggles to stay up to date. Yet, half of the midwives felt overwhelmed by all the digital developments in their field, which prevented them from implementing mHealth apps in practice. Nevertheless, most midwives were optimistic about the prospects of apps, and did intend to implement mHealth apps in their standard care on the condition that the app was easy to use and trustworthy. 

Most midwives in our study provided individual face-to-face nutritional counselling that was limited to general dietary recommendations for pregnant women, and referral to the Dutch Nutrition Centre; personalised nutritional counselling was considered to be the expertise of a dietician. In line with our findings, De Jersey et al. [32] also concluded that the nutritional knowledge level of professionals was poor, and their advice limited. Correspondingly, Basu et al. [33] developed and evaluated a nutritional training for midwives. De Jersey et al. [32] and Basu et al. [33] both raised the benefit of some additional nutritional training for midwives to facilitate identification of nutritional inadequacies. However, clearly, there are boundaries to the level of nutritional counselling by midwives, which again highlights the need for more personalised nutritional mHealth tools to support these professionals. 

In our study, most midwives showed an open and positive attitude on the use of mHealth as part of their counselling. However, some midwives were reluctant and/or unfamiliar with the use of mHealth apps and indicated that information provision on this topic through the Dutch professional network KNOV was minimal. These findings add to recent findings of a 23-question survey by Vasiloglou et al. [34] completed by 1001 professionals, mostly female medical doctors, with a mean age of 34 (SD 10) years from 73 countries all over the globe showing that 46% of the professionals recommended nutritional mHealth apps to their patients. Of the 54% of the professionals that did not recommend the use of these apps, 23% was not aware of their existence. Additionally, a recent qualitative study among professionals, including twelve in-depth interviews, indicated that professionals’ lack of familiarity with and fear of mHealth limited the use mHealth to support nutritional counselling during pregnancy [35]. To illustrate, some professionals felt that the implementation of mHealth could lead to a shift from trusted sources, such as health professionals and health organisations, to less trustworthy sources [35]. In line with our results, professionals suggested that funds, mHealth literacy, commercially developed apps, and lack of advocacy could also be potential barriers to recommend app use [35]. Moreover, similar to the midwives in our study, professionals indicated that an app should be easy to use (87%), self-explanatory (51%), and validated (69%) in order for them to recommend use of such an app [34]. In line with these findings, Verkasalo et al. [36] summarised earlier research on the general use of apps and concluded that efficiency, simplicity, and pleasure are key predictors of the continuous use of apps by end-users. A framework for the evaluation of mHealth apps was proposed in a more recent study and stated that attractiveness, value, ease-of-use, trust, social support, diffusiveness, fun, and excitement are important influences to use an app [37].

Although a systematic review acknowledged the potential of mHealth apps to support healthcare [21], current evidence on the effectiveness of these apps is limited, which may also explain their limited use in practice. Dennison et al. [38] also acknowledged the challenge of designing an effective app and stated that it is imperative to incorporate appropriate and attractive behavioural change strategies while limiting users’ registration burden. Registration burden is a commonly known issue with personalised nutritional coaching tools, as self-reported recall or record methods are most used to assess food and nutrient intake, which are in turn time-consuming to complete and often considered burdensome by users [39]. In addition, to provide personalised nutritional counselling, it is eminent to adopt behaviour change theory to identify and influence key constructs related to behaviour change [40]. Currently, the integration of behaviour change theories in nutritional coaching apps is rather limited, which could explain the limited success of these apps [41,42].

Thus, developing a high-quality nutritional coaching app for pregnant women will be challenging, and integrating behaviour change theories, usability, perceived benefit, and trustworthiness seem crucial aspects for both pregnant women to change behaviour and midwives to advise such apps. It is therefore of utmost importance to address the highlights that have emerged from scientific studies so far in the development of new apps. This should already start by involving stakeholders (e.g., midwives, GPs, obstetricians, dieticians, potential end-users) at an early stage of the developmental process [43]. This will not only benefit the app’s quality, but also ensures that the app aligns with the needs and preferences of professionals and end-users, and thus the likelihood of professionals’ implementing the app in daily practice [37,44]. Content-wise, midwives in our study recommended developing an interactive app with features of a dietary diary with automatically integrated feedback, personalised push notifications, recipes, and fun facts about the health of mother and child related to the phase of the pregnancy.

Although this study was the first to explore the perspectives and experiences of Dutch midwives on this topic, our findings cannot be evaluated without considering the following methodological aspects. First, it may be seen as a limitation that all participants in this study were women. However, a mixed sample of men and women was not expected as most midwives in the Netherlands are women. Still, this could have limited the generalisability of our finding to all Dutch midwives. Nonetheless, we do consider the types of midwives participating in this study to be diverse, i.e., there is a large age-range, midwives come from geographical locations from all over the Netherlands, midwives executed their profession in either a hospital setting or community care, and midwives come from rural as well as urban areas. Furthermore, pro-innovation bias, meaning that the value of the usefulness of mHealth apps might be overrated, could have affected this research due to the pro-innovation attitude of the researchers. A strength of this study is that it was conducted through in-depth interviews and according to the integrated TDF-COM-B framework, which has been used before to explore the experiences and preferences of midwives [45]. Additionally, member checks were performed to assure the validity of the main message of the interviews. Finally, all transcripts were coded by two researchers under the supervision of a senior researcher to assure accuracy and reduce bias.

## 5. Conclusions

We explored the experiences and perspectives of midwives on online (nutritional) counselling and the use of mHealth apps for pregnant women to contribute to the development of dietary mHealth tools for pregnant women. We observed that Dutch midwives only refer clients to the ZwangerHap app from the Dutch Nutrition Centre. Trustworthiness, user-friendliness, and understandability were key factors in the considerations on whether to refer to mHealth apps. Moreover, the interviews suggested that the involvement of midwives and other professionals in the design of the app could be an important aspect to design an app meeting the demands of professionals and end-users. However, due to limited knowledge and time on the subject matter by midwives, there is a need for guidelines and standard practices on how to refer clients to suitable apps. Accordingly, future and current apps should be well communicated through professional networks to achieve visibility and trust by informing midwives about the purpose, target population, usability, and scientific background of the tool. Overall, midwives were optimistic about the future of apps and think this can enhance the health of mother and child.

## Figures and Tables

**Table 1:** Characteristics of participants.

Participant Number	Age in Years	Workplace	Employment	Work Area
P1	38	Primary care	Full time	Rural
P2	53	Hospital	Full time	Urban
P3	27	Primary care	Full time	Rural
P4	35	Hospital	Full time	Urban
P5	22	Primary care	Full time	Urban
P6	60	Primary care	Full time	Urban
P7	21	Primary care	Full time	Urban
P8	29	Combined	Full time	Urban
P9	23	Primary care	Full time	Urban
P10	25	Primary care	Part-time	Urban
P11	49	Primary care	Part-time	Rural

## Data Availability

The anonymous datasets generated and/or analysed during the cur-rent study are available from the corresponding author on reasonable request.

## Supplementary Materials

The following are available online at https://www.mdpi.com/article/10.3390/ijerph18136733/s1, Interview Guide S1.

## Appendix A

**Table A1:** Codes and sub-codes.

Codes	Sub-Codes
Current nutritional advice	Pre-conceptional consult When nutritional advice Pregnant women’s interest in nutrition
	Emphasis nutritional advice Nutritional knowledge Referral to dietician
	Non-mHealth sources Nutritional apps
	Expected changes nutrition
Experiences with digitalisation	Role professionals’ association Experiences online counselling Experiences digital advice vs. personal advice COVID-19
	Updates (nutritional) innovations Flexibility and age-related acceptance of innovations
	Referral to apps Knowledge of apps
	Vision digital counselling
Potential uses for mHealth apps	Opinion use of pregnancy app
	Features apps
	Role midwives in app-use
	Role of apps in the future
