# Supplementary material for: Midwives’ Experiences with and Perspectives on Online (Nutritional) Counselling and mHealth Applications for Pregnant Women; an Explorative Qualitative Study
Source: Int J Environ Res Public Health. 2021 Jun 23;18(13):6733. doi: 10.3390/ijerph18136733 (PMC8267613; doi:10.3390/ijerph18136733)
Supplement: Supplementary file 1 [file ijerph-18-06733-s001.zip › ijerph-1225700-supplementary.pdf]

International Journal of
Environmental Research and Public Health

MDPI

Midwives' Experiences with and Perspectives on Online (Nutritional) Counselling and mHealth Applications for Pregnant Women; an Explorative Qualitative Study

Factsheet interview

Participant number Deelnemersnummer	
Date and time<br>Datum en tijd	
Age<br>Leeftijd	
Work area<br>Werkgebied	
Other circumstances (e.g. temporary inactivity/digital consultations due to corona)<br>Andere omstandigheden (bijv. tijdelijk niet werkzaam/digitale consulten ivm corona)	

Topic List

What do you think about online counselling or coaching?
Hoe denk je over online begeleiding of coaching?
What do you think of the 'health apps' that are available nowadays?
Wat vind je van de 'gezondheidsapps' die tegenwoordig beschikbaar zijn?
How do you think these kinds of apps will play a role in the future?
Hoe denk je dat dit soort apps een rol spelen in de toekomst?
What would you like with more insight into your diet?
Wat zou je willen met meer inzicht in het voedingspatroon?

(1) Knowledge

Do you know which apps are available?
Weet u welke apps er beschikbaar zijn?

(2) Skills

Are you able to advice useful apps?
Bent u in staat om nuttige apps te adviseren?

(3) social/professional role and identity

How do you feel about your position as a healthcare professionals regarding nutritional advices?
Wat vindt u van uw positie als zorgverlener op het gebied van voedingsadviezen?

(4) beliefs about capabilitie

Do you feel capable enough to provide pregnant women with advices about apps for nutritional coaching or refer them to e.g. a dietician?

Voelt u zich in staat om zwangere vrouwen advies te geven over apps voor voedingscoaching of hen door te verwijzen naar bijv. een diëtist?

(5) optimism

Do you feel optimistic about the potential influence of online nutritional coaching for pregnant women?

Voelt u zich optimistisch over de mogelijke invloed van online voedingscoaching voor zwangere vrouwen?

(6) beliefs about consequences

Do you believe your action regarding nutritional (online) coaching have consequences for your or your patient/client?

Gelooft u dat uw actie op het gebied van nutritionele (online) coaching gevolgen heeft voor uw of uw patiënt/klant?

(7) reinforcement

How do you feel about arranging a dependent relationship with a patient/client where you can encourage or ‘punish’ the patient when agreements are not performed?

Wat vindt u van het regelen van een afhankelijke relatie met een patiënt/klant waarbij u de patiënt kunt aanmoedigen of 'straffen' als afspraken niet worden nagekomen?

8). intentions

Do you have the intention to stimulate pregnant women to improve their nutritional intake of even have this conversation about nutrition?

Heeft u de intentie om zwangere vrouwen te stimuleren om hun voedingsinname te verbeteren of zelfs dit gesprek over voeding te voeren?

(9) goals

How do you feel about setting yourself a goal for your intentions to improve dietary intake in pregnant women or the use of an app to improve this?

Wat vind je ervan om jezelf een doel te stellen voor je intenties om de inname van voeding bij zwangere vrouwen te verbeteren of het gebruik van een app om dit te verbeteren?

(10) Memory, attention and decision processes

Are you informed enough to make a decision for the patient about which advice to give concerning nutritional advices or apps?

Bent u voldoende geïnformeerd om voor de patiënt een beslissing te nemen over welk advies hij of zij moet geven over voedingsadviezen of -apps?

(11) environmental context and resources

Do you feel to have all resources available to do e.g. online coaching?

Heeft u het gevoel dat u alle middelen beschikbaar heeft om bijvoorbeeld online coaching te doen?

(12) social influences

What are social influences which effect your behaviour and opinion on online (nutritional) coaching?

Wat zijn sociale invloeden die je gedrag en mening beïnvloeden op online (voedings)coachen?

(13) emotion

What emotions do you feel regarding the subject nutritional coaching? And what about online coaching?

Welke emoties voel je met betrekking tot het onderwerp voedingscoaching? En hoe zit het met online coaching?

(14) Behavioural regulation

How do you feel about the flexibility of your own behaviour when it comes to improved technology in the form of apps for pregnant women?

Wat vindt u van de flexibiliteit van uw eigen gedrag als het gaat om verbeterde technologie in de vorm van apps voor zwangere vrouwen?

Post-interview comment sheet

Express comments after the interview
